# The Role of Grit in Organizational Performance During a Pandemic

**DOI:** 10.3389/fpsyg.2022.929517

**Published:** 2022-07-07

**Authors:** Joonghak Lee

**Affiliations:** College of Business, Gachon University, Seongnam, South Korea

**Keywords:** grit, supportive climate, transformational leadership, organizational performance, personal trait in crisis

## Abstract

In the context of the organizational crisis caused by COVID-19, scholars and professionals have focused on factors that help employees stay at their jobs and perform well. In an uncertain era, grit is a significant employee and organizational sustainability trait. Using 890 responses, this study determined how grit affects organizational performance and used contingencies including supportive climate and transformational leadership as moderators. The hypotheses were tested by examining the relationship between grit and organizational performance and the moderating effects of supportive climate and transformational leadership. Further, these hypotheses were supported by confirmatory factor analysis, PROCESS macro analysis, and bootstrapping. Grit was found to be positively associated with organizational performance; meanwhile, supportive climate and transformational leadership strengthen the relationship between grit and performance. Both theoretical and practical implications of the findings are discussed. This study makes a theoretical contribution through its assessment of the impact of grit on organizational performance. Trait activation theory can explain how grit can be expressed through organizational climate and leadership. With regard to practice, grit can be used as a vital factor for personnel selection and a supportive climate should be provided to ensure a desirable organizational climate.

## Introduction

The business environment of an organization is constantly changing, and the competition for survival is fiercer than ever (Fernández-Aráoz, 2014). On March 11, 2020, the World Health Organization declared the COVID-19 outbreak a pandemic; it is recognized as becoming the fastest and most widely spreading disease since the 1918 influenza pandemic (Ferguson et al., 2020). This public health crisis has resulted in simultaneous decreases in demand and supply (Gopinath, 2020) and influenced employee attitudes and behaviors at work (Nguyen et al., 2022). Further, companies are facing unprecedented organizational crises (Budhwar and Cumming, 2020). Organizational crises are defined as time-consuming and uncertain events or time periods caused by specific issues (Pearson and Clair, 1998). An organizational crisis causes anxiety and despair among members, teams, and organizations as a whole and also in terms of employee turnover and financial losses (Madera and Smith, 2009). During a crisis, organizations recognize that the environment is highly uncertain, and employees are expected to constantly perceive uncertainty in their daily work environment (Mitroff et al., 1988). Thus, what are the essential qualities for employees to work effectively during such a crisis?

Personal resources such as motivation, traits, and characteristics aid individuals under difficult circumstances (Sinclair, 2017). Grit is among the most well-known factors that may increase employees' adaptability to create a thriving environment (Arco-Tirado et al., 2019). In an uncertain environment, it is critical to maintain one's passion and perseverance (Littman-Ovadia and Lavy, 2016; Luthans et al., 2019). Grit can be defined as “the passion and perseverance for achieving long-term goals” (Duckworth et al., 2007, p. 1087). Individuals must be tenacious to keep up with environmental changes (Westphal et al., 2008). Thus, scholars and professionals worldwide and industries have paid close attention to grit (Jordan et al., 2019).

Despite its widespread acceptance as a predictor and determinant of performance, more research is needed to better understand the nature and mechanism of grit (Credé et al., 2017; Khan et al., 2020). There has been little research that sheds light on how each employee's grit is activated in response to their environment (Jordan et al., 2019; Choi et al., 2020). Previous research on grit at the individual level has yielded conflicting results (Kannangara et al., 2018). For example, while one study found that women have higher levels of grit than men on average (Schmidt et al., 2018), another found no difference between women and men in terms of grit (Hodge et al., 2018). Similarly, some studies have failed to investigate the positive impact of grit on achievement-related variables (Lam and Zhou, 2022).

This study aims to determine how grit can improve organizational performance by discussing factors such as supportive climate and transformational leadership. Previous research has shown that employees with higher grit are more motivated to perform well. This study presents a model of the relationship between grit and organizational performance that includes two moderators. It adds to the literature by proposing contingencies and psychological mechanisms based on trait activation theory (TAT), addressing mixed results of the impact of grit. Second, this study focused on macro-level performance, which has not been discussed much in previous studies. Third, we contribute to the literature by indicating the personal characteristics required in a crisis-stricken organization.

Furthermore, HR managers can consider grit as a significant trait for personnel selection and improve the supportive climate to boost organizational performance through gritty employees. When a gritty employee contributes to the organization, supportive climate and transformational leadership can boost grit's impact. From the practitioner's perspective, transformational leadership is an important characteristic and style for increasing the impact of grit in the organization. Furthermore, HR managers can promote and develop leaders with such characteristics through training and coaching programs, indicating specific implications for the HR department.

The remainder of this paper is organized as follows. A literature review and overarching theory were used to develop hypotheses. Thereafter, sections on methodology, results, discussion, and conclusion are presented.

## Literature Review and Hypothesis Development

### Trait Activation Theory

The TAT has contributed to studies explaining how individual traits, that is, predispositions of employees to behave consistently in response to situational stimuli, are an important determinant of individual behavior in the workplace (Day and Silverman, 1989). TAT shows that the impact of a trait would vary depending on the cues provided by work situations (Tett et al., 2021). TAT emphasizes situation trait relevance to understand where a personality trait is most likely to manifest itself in conduct (Tett and Guterman, 2000) and posits that traits interact with trait-related situational activators (O'Brien et al., 2021). The importance of situational strength in TAT cannot be overstated.

Situation strength refers to the extent to which situational limitations are prevalent in the environment (Lievens et al., 2006). Strong situations have unambiguous behavioral demands, with the consequences of behavior clearly identified and discussed (Caspi and Moffitt, 1993). In contrast, in weak situations, behavioral expectations are relatively ambiguous, resulting in huge differences in how individuals respond to situations.

TAT proposes three sources of trait-relevant cues: task-related cues (e.g., daily activities), social cues (e.g., interactions with coworkers), and organizational cues (e.g., organizational climate, culture, and structure; Mischel, 1973). The importance of a trait and its context should be aligned such that the individual possesses the trait that allows them to respond effectively to the situation's indications (Luria et al., 2019). By stating the following, the trait activation principle formalizes the trait–situation relationship: Arousal of a trait by a trait-relevant situational stimulus is required for the behavioral expression of that trait (Tett and Guterman, 2000).

### Grit

Grit has received much attention as a crucial characteristic for career success (Clark and Clark, 2019). It has been identified as a predictor of personal achievement (Duckworth et al., 2007), and researchers have recently highlighted the importance of grit for goal achievement in the face of adversity and challenge (Jordan et al., 2019). Traits are personal differences that reflect relatively stable temperaments of thought, feeling, or behavior (Fleeson and Jayawickreme, 2015). Behavioral traits reveal how people react (Matthews, 2018). For nearly a century, psychologists have debated whether consistent effort and focused interest are distinct from talent but equally important to success (Eskreis-Winkler et al., 2014). The number of studies on grit has increased, but none have presented consistent results in terms of performance (Lam and Zhou, 2022). Grit, an unrecognized characteristic of individuals that leads to success in today's era, is gaining attention because it can be developed (Lee and Stankov, 2018) and has a significant impact on individual achievement, including organizational citizenship behavior and academic performance (Heckman and Masterov, 2007; Arifin et al., 2019; Luthans et al., 2019).

Grit can be a psychological resource that boosts peak performance in different settings and contexts (Duckworth and Quinn, 2009). For example, a person with high grit excels in a variety of activities (Eskreis-Winkler et al., 2014). Employees with high grit are interested in long-term goals and strive to achieve them despite internal and external obstacles. A recent meta-analysis discovered a link between grit and performance (Credé et al., 2017).

Grit is defined as a desire to focus on a specific goal and the persistence to strive for it despite failures and adversity (Duckworth et al., 2007). Gritty employees continue to practice deliberately to gain new skills (Duckworth et al., 2007; Jordan et al., 2019). Employees are expected to cope and survive in a workplace where uncertainty and complexity are increasing (Luthans et al., 2019). However, they are likely to face difficulties in their work, which increases the likelihood of failure. Grit as a personal trait provides the resilience required to persevere in such situations (Gilson and Davis, 2019). According to TAT, if the organization is made up of hardworking employees, the firm can expect high performance in the event of a crisis. Accordingly, we hypothesize the following:

*Hypothesis 1. Grit is positively related to organizational performance*.

### Supportive Climate

Several decades of research have revealed that organizational climate has a significant impact on critical economic outcomes (e.g., Schneider et al., 1998, 2011; Borucki and Burke, 1999; Collins and Smith, 2006). Psychological climate can be defined as current impressions of events, practices, and processes and the types of behaviors that are rewarded, supported, and expected in a certain environment (Schneider, 1987; James et al., 2008). Organizational climate can be defined as a reflection of the behaviors and reactions of employees to what the organization assumes and emphasizes (Khalili, 2016). Further, employee perceptions of processes, policies, and practices are referred to as organizational climate (Reichers and Schneider, 1990).

Organizational climate performs a directive function by channeling employee behaviors toward critical organizational goals (Slåtten et al., 2021). A climate exists when the significance of environmental events and traits is sufficiently shared by a large group of people, such as a workgroup or business unit (McKay and Avery, 2006; Zhao et al., 2019). Moreover, the climate develops because of organizational rules and processes that promote specific employee actions and behaviors, resulting in the formation of shared behavioral expectations (Bowen and Ostroff, 2004).

Individual performance can be explained as a multiplication of ability, support, and effort (Schermerhorn et al., 1990). Simply put, employees can perform based on the support they receive from their organization, colleagues, and systems. Renn and Prien's (1995) study first introduced the connection between a supportive climate and performance. Research shows that a positive organizational climate is associated with higher performance (Gardner III and Schermerhorn, 1992; Ferris et al., 1998; Gardner and Schermerhorn, 2004). A supportive climate is defined as how members trust, care for, and collaborate with one another, and it is an important type of psychological climate in the organization (Kim et al., 2021). The study created a supportive climate by focusing on desirable outcomes, such as performance, job satisfaction, and commitment, and demonstrating construct validity (Rogg et al., 2001; Tripathi and Tripathi, 2022).

Because TAT suggests that organizational climate can activate personal traits, we propose that perceptions of a supportive climate may create the strong environment required for grit to thrive. The organizational climate and operational levels can serve as cues to activate personal traits (Tett et al., 2021). Individuals, for example, are more likely to face new challenges and strive to overcome them if they perceive support from their organizations and colleagues. In other words, a positive work environment can act as a trait activator in the relationship between grit and perceived organizational performance. The following hypotheses are established based on prior studies and inferences:

*Hypothesis 2. A supportive climate strengthens the relationship between grit and organizational performance*.

### Transformational Leadership

Transformational leadership has received considerable attention from scholars and practitioners (Charoensukmongkol and Puyod, 2021; McCombs and Williams, 2021; Kloutsiniotis et al., 2022) because organizations face challenges and high unpredictability (Santoso et al., 2021). When an organization undergoes rapid change, the leader must demonstrate a clear direction and a shared vision for organizational change (Atkinson and Mackenzie, 2015). Transformational leaders present a vision for the future in order to change the organization and elicit members' participation to achieve it (Pawar and Eastman, 1997; Popli and Rizvi, 2017; Park et al., 2019). Further, they use positive emotions and messages to present their vision and motivate employees toward higher performance (Bass, 1985, 1990; Shamir et al., 1993). The transformational leadership construct consists of four components: “idealized influence,” “intellectual stimulation,” “inspirational motivation,” and “individualized consideration” (Bass and Avolio, 2000). Transformational leaders influence their followers in two ways: first, by developing employees (Bakker et al., 2022) and, second, by strengthening the relations between the employee and the organization (Cole et al., 2009).

Transformational leaders assist employees in producing better-than-expected results (Kouzes and Posner, 1996; Epitropaki and Martin, 2005). Further, transformational leaders, in particular, focus on supporting employees, enabling them to have a bond with their organization *via* effective internal communication (Santoso et al., 2022). TAT emphasizes the context dependence of the relationship between traits and performance (Luria et al., 2019). A study conducted in 78 countries and 22 industries showed a positive relationship between grit and transformational leadership (Caza and Posner, 2021). Social cues, such as leadership, activate latent traits (Tett and Guterman, 2000). Therefore, we propose the following hypothesis:

*Hypothesis 3. Transformational leadership strengthens the relationship between grit and organizational performance*.

## Method

### Data Collection and Common Method Bias

This study conducted an online survey of employees at company A, a prominent South Korean commercial conglomerate active in the retail, food, service, hotel, chemical, and other industries. It now has a workforce of more than 55,000 local employees and 400 expatriates from 31 countries. We chose company A as our data source because it is among the most recognizable companies in South Korea considering its diverse industry offerings. We met a manager from company A's human resources department and asked him to randomly select samples from local employees of 36 affiliates to avoid convenience sampling bias. The authors were given a list of potential participants based on gender and age derived from the HR system but without personal identifiers or e-mail addresses. Finally, the poll was sent to 2,400 employees using the company's internal mail system, which took 15 mins. Among 1,600 local employees, 890 completed the survey (56% response rate). The reason behind the low response rate might be because of the anonymity ensured in the survey to avoid common method bias (Podsakoff et al., 2003).

According to Baruch and Holtom (2008), the average response rate of 463 studies was 52.7%, and they argue that response rate is a factor to consider when assessing the quality of empirically conducted research. Therefore, because the response rate was low in this study, we have identified it as a weakness in the limitation section. The participants belonged to 36 companies. Their job ranks were *manager* and *senior manager*. Moreover, the female and male ratios were 30.2 and 69.8%, respectively. The participants belonged to the following age groups: 20–29 years (22.2%), 30–39 years (37.0%), 40–49 years (30.4%), and 50 years and above (10.3%). Because our sample is biased toward males, we consulted previous literature on gender differences for grit and controlled for gender in the analysis. Some studies have found gender differences in grit (Rojas et al., 2012; Christensen and Gerald, 2014), while others have found no differences (Batres, 2011; Washington, 2016; Hodge et al., 2018).

Furthermore, our data came from a single source, which poses a risk of bias in behavioral research (Rodríguez-Pinto et al., 2011). We conducted the survey anonymously, improved the readiness of item wording, and separated the measurement of the independent and dependent variables, as recommended by Podsakoff et al. (2003). We also used Harman's one-factor analysis, and the results showed that all measures of goodness of fit for the one-factor model were worse for the original measurement model data (**χ^2^**/df = 14.91, CFI = 0.72, TLI = 0.70, RMSEA = 0.12, SRMR = 0.10). Therefore, common method bias was deemed unproblematic with this dataset.

### Measurement

The explanatory variable (grit), two potential moderating variables (supportive climate and transformational leadership), and response variable (organizational performance) were all observed, along with demographic and other variables (gender, age, and job rank). Except for the control variables, all variables were evaluated using a 6-point Likert scale (1 = not at all, 6 = very likely), and the reliability of the variables was confirmed using the Cronbach's alpha index.

#### Grit

Duckworth and Quinn (2009) proposed the Short Grit Scale (Grit–S) for measuring grit. Furthermore, the sub-elements passion and provenance were each measured with four questions; thus, the scale had a total of eight questions. This scale is a simplified version of the Grit-O (Duckworth et al., 2007), which is comprised of 12 questions. “When a new project is created, I often feel that it comes in the way of existing work,” “I find it difficult to see my pre-set goals through till the end,” “I do my best as much as much as possible” and “I do not easily get frustrated even if there are obstacles,” are some sample questions from the instrument. The Cronbach's alpha, which indicates the scale's internal reliability, was 0.74.

#### Supportive Climate

To assess the supportive climate (*SC*), we used items reported by Rogg et al. (2001) that had shown significant validity in previous research (Luthans et al., 2008). We used five items from two of the four factors to create a shortened scale that included aspects of SC that were most relevant to their research (i.e., employee cooperation and coordination factors). Examples of items include “My organization collaborates to get the task done effectively and efficiently” and “Employees make personal sacrifices when necessary to help the firm prosper.” The Cronbach's alpha of the scale was 0.90.

#### Transformational Leadership

Transformational leadership (*TL*) was examined by translating Bass and Avolio's (1993) MLQ-6S. TL consists of four components, and each factor is measured using four items. Examples of the items include “My CEO acts to make employees feel good about being around him/her,” “My CEO can clearly express what the company can do and will be able to do,” and “My CEO lets employees think of the problems they have solved so far in a new way and seem them in a new perspective.” The Cronbach's alpha of the scale was 0.95.

#### Organizational Performance

This study used the variable *perceived market performance* to assess organizational performance (*OP*) (Singh, 2004). Because it was derived from the question presented to participants to evaluate their company's OP by comparing it to competitors, the measure was relative or benchmarked. This variable included sales growth, profitability, market share, and the quality of the product or service. A sample item is “How would you rate your company's sales growth in comparison to its main competitors?” The Cronbach's alpha of the scale was 0.90.

#### Control Variables

Three variables including gender, age, and job rank were used as control variables based on previous research. Some studies have reported a positive relationship between grit and achievement-related variables, so we controlled for age in the analysis (Rojas et al., 2012; Christensen and Gerald, 2014). Duckworth et al. (2007) found that grit is stable over time, but recent research has shown that grit levels can increase with age (Cosgrove et al., 2016; Peña and Duckworth, 2018). Because higher positions in the organization, including leadership, can have a positive relationship with grit during difficult times, job rank was used as a control variable (Rego et al., 2021). Strong situations may compel leaders to take specific actions (Meyer et al., 2010), and it may cause leaders to demonstrate grit (Rego et al., 2021). In that sense, employees in higher positions may demonstrate a higher level of grit because the data was collected during a pandemic and then controlled in the analysis.

### Analytic Procedure

For descriptive purposes, among the four variables, the Pearson's correlations were estimated, and the mean and standard deviation of each variable were calculated by age, gender, and job rank. We used Amos 21.0 to perform a confirmatory factor analysis (CFA) before testing our hypotheses to assess the relevance of the research variables and check for common method bias. When the relationship between variables and factors is not theoretically established or logically organized, exploratory factor analysis is used to investigate the structure and reduce the number of variables to increase statistical efficiency (Hurley et al., 1997). CFA, on the other hand, assumes that specific measurement variables are necessarily affected by related (latent) variables and are unrelated to other factors based on a strong theoretical background or previous studies (Stapleton, 1997). Thus, CFA is similar to theory testing and has a theory-driven nature. As a result, we used CFA to test hypotheses and examine variables' structure.

**Figure 1:**
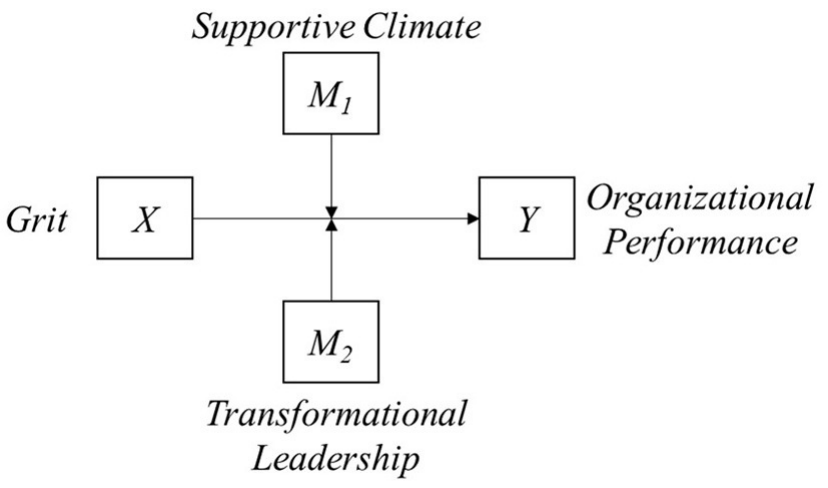
Research model.

To address the study objective, we conducted the moderation analysis using two potential moderators: SC and TL (Figure 1). Hayes (2012) refers to this as Model 1. According to Hayes et al. (2017), organizational behavior researchers used PROCESS macro in the empirical literature, discovered a trivial difference from structural equation modeling, and presented the moderation effect verification method. Therefore, the PROCESS macro was used in this study to test our hypotheses. Moreover, this study used bootstrapping to investigate the significance of the indirect effects using SPSS 21.0 macro model module to obtain the upper- and lower-class boundaries with 95% confidence level along with coefficients and standard errors (Islam et al., 2022).

## Results

### Correlation and CFA

Table 1 displays descriptive statistics for the variables studied (i.e., mean, standard deviations, and correlation coefficients). Except for the relationship between job rank and other variables, all variables are positively related.

**Table 1:** Estimated correlations among grit, OP, SC, TL, and demographic information scores.

**Construct**	**M**	**SD**	**1**	**2**	**3**	**4**	**5**	**6**
1. Grit	4.60	0.54	–					
2. OP	3.81	0.88	0.27^**^	–				
3. SC	4.39	0.85	0.47^**^	0.49^**^	–			
4. TL	4.59	0.86	0.45^**^	0.48^**^	0.58^**^	–		
5. Age	2.29	0.93	0.26^**^	0.13^**^	0.29^**^	0.38^**^	–	
6. Gender	1.70	0.46	0.18^**^	0.09^*^	0.18^**^	0.23^**^	0.36^**^	–
7. Job Rank	1.96	1.16	−0.03	−0.09	−0.07	−0.06	−0.03	0.09

*OP, organizational performance; SC, supportive climate; TL, transformational leadership*.

*N = 890;*

* *p < 0.05*,

** *p < 0.01*.

Then, we conducted CFA using AMOS 21.0 to check the validity of the variables in this study (Ahmad et al., 2021; Islam et al., 2022). Table 2 shows the CFA results. Overall, our research models show acceptable fit (CFI = 0.92, TLI = 0.91, SRMR = 0.05, RMSEA = 0.08; CFI = 0.93, TLI = 0.93, SRMR = 0.04, RMSEA = 0.07) compared to alternative models. The findings show that the measurements are discriminately valid and that common method bias is unlikely.

**Table 2:** Confirmatory factor analysis.

**Construct**	**χ^2^/df**	**CFI**	**TLI**	**SRMR**	**RMSEA**
Three-factor model^a^	6.64	0.92	0.91	0.05	0.08
Three-factor model^b^	5.24	0.93	0.93	0.04	0.07
Two-factor model^c^	12.06	0.79	0.78	0.09	0.11
One-factor model^d^	14.91	0.72	0.70	0.10	0.12

*χ^2^/ df, chi-square/degree of freedom; CFI, comparative fit index; TLI, Tucker–Lewis index; SRMR, standardized root mean square residual; RMSEA, root mean square error of approximation*.

a *Grit + SC + OP*,

b *grit + TL + OP*,

c *grit, SC + TL + OP*,

d *all four factors were combined into a single factor*.

### Hypothesis Test

Multiple linear regression was used to test hypothesis 1, which states that grit and OP are positively related. The first block included control variables (i.e., job position, gender, and age) and the second included grit. Grit is related to OP (β = 0.25, *p* = 0.001; Table 3), which supports hypothesis 1.

**Table 3:** A regression-based path analysis for the moderation model of supportive climate (SC) and transformational leadership (TL).

**Variable**	**Model 1**	**Model 2**	**Model 3**	**Model 4**	**Model 5**	**Model 6**
**Control variable**						
Job	−0.10^*^	−0.09	0.07	−0.06	−0.07	−0.07
Gender	0.06	0.04	0.01	0.01	0.01	0.00
Age	0.01^*^	0.05	−0.03	−0.03	−0.07	−0.08
**Independent variable**						
Grit		0.25^**^	0.06	−0.25	0.08^*^	−0.35^*^
Moderators						
SC			0.46^**^	−0.07		
TL					0.46^**^	−0.25
Interacting variable of SC				0.74^*^		
Interacting variable of TL						0.98^**^
Adjusted R^2^	0.02	0.08	0.23	0.24	0.23	0.24
R^2^	0.03	0.09	0.24	0.25	0.24	0.25
F	8.277^**^	20.572^**^	56.490^**^	48.245^**^	55.418^**^	48.193^**^

*N = 890;*

* *p < 0.05*,

** *p < 0.01*.

**Figure 2:**
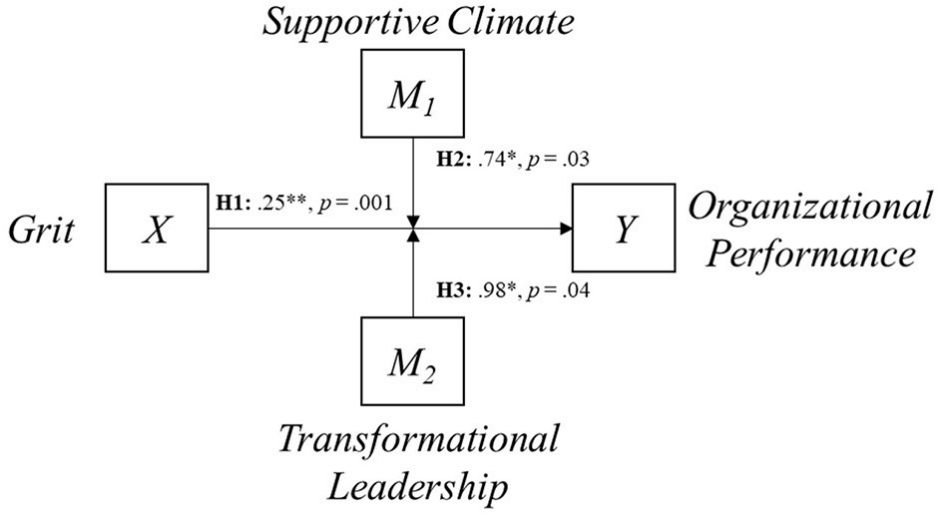
Path relationship for the moderation model. *p < 0.05, **p < 0.01.

To test hypothesis 2, the moderating effect of a SC on grit and OP, the PROCESS macro, which was designed to evaluate multiple mediation and moderation models, was used (Choi et al., 2020). We used Model 1 (Process macro) and an additional 95% bias-corrected confidence interval with 5,000 bootstrapping procedures to investigate the moderation effects of SC and TL because 5,000 is sufficient for a robust analysis (Mundform et al., 2011). Table 3 and Figure 2 show that SC and TL strengthen the relationship between grit and OP (β = 0.74, *p* = 0.03; β = 0.98, *p* = 0.04), thus supporting hypotheses 2 and 3.

As in Table 4, the indirect effects of SC and TL are suggested with the results (BootSE = 0.06, Boot 95% CI = [0.00, 0.21]; BootSE = 0.05, Boot 95% CI = [0.03, 0.24], respectively), which confirm the significance of moderation effects (Preacher and Hayes, 2004) because the results do not include any zero value between the upper- and lower-class boundaries (Byrne, 2010).

**Table 4:** Conditional indirect effects.

	**Conditional** **indirect effect**	**Boot SE**	**LL 95% CI**	**UL 95% CI**
**Supportive climate**				
High grit (+1SD)	0.02	0.07	0.01	0.21
Low grit (−1SD)	0.19	0.07	0.05	0.33
**Transformational leadership**				
High grit (+1SD)	0.02	0.07	−0.11	0.15
Low grit (−1SD)	0.28	0.08	0.13	0.43

*N = 890, Bootstrapping times = 5,000*.

**Figure 3:**
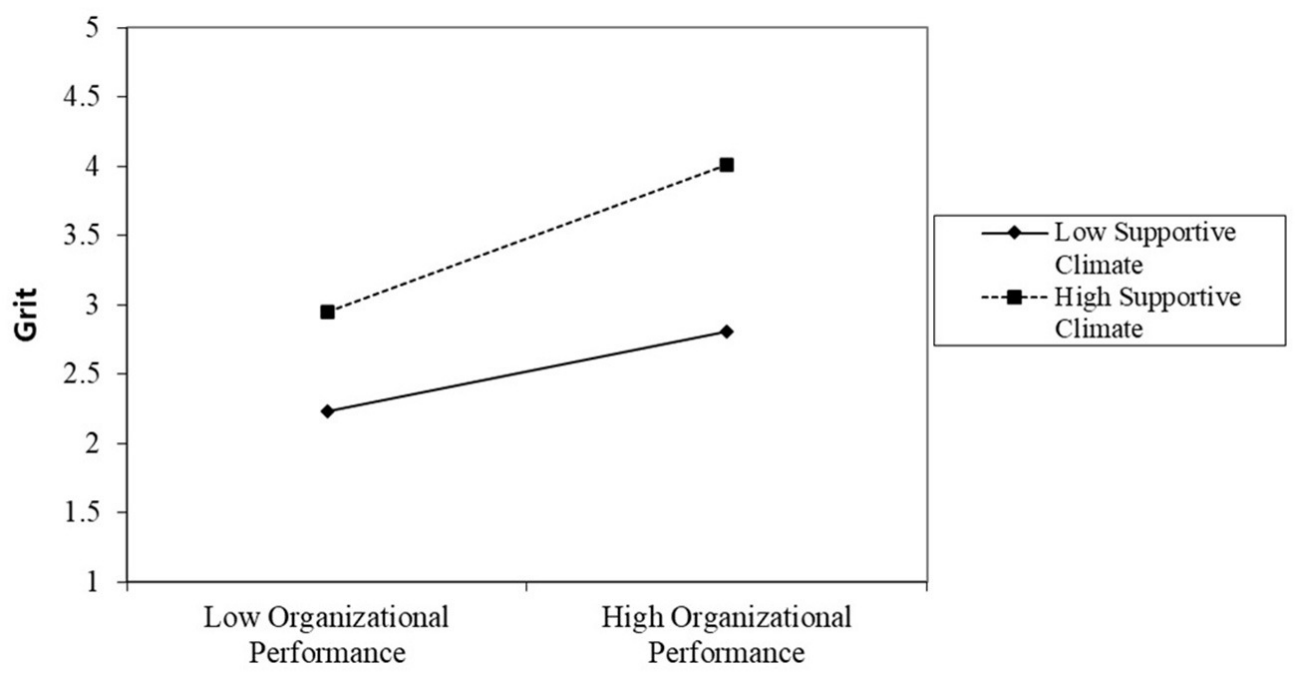
Moderating effect of supportive climate on the relationship between grit and organizational performance.

**Figure 4:**
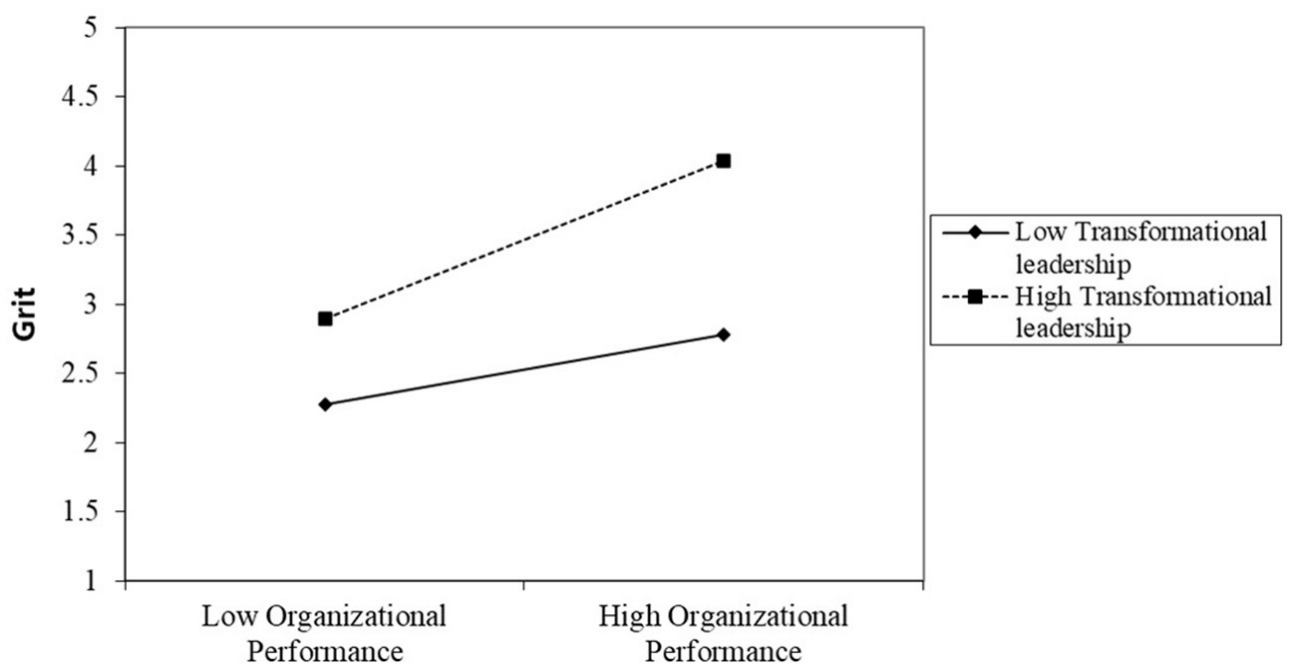
Moderating effect of transformational leadership on the relationship between grit and organizational performance.

To investigate the interaction effect, we used simple slopes, as recommended by Aiken et al. (1991). If the interaction terms of the independent and moderating variables show a significant relationship, the graph of the interaction must be schematically drawn using the mean value and ±1 standard deviation, and a simple slope test must be performed (Aiken et al., 1991). A simple slope must be computed to clarify the form of the moderating effect (Jaccard et al., 1990). The positive relationship between grit and OP becomes stronger as SC and TL increase (see Figures 3, 4). In other words, SC and TL can help strengthen the bond between grit and OP.

## Discussion

Employees with a higher level of grit can perform their best in a variety of situations (Duckworth and Quinn, 2009). However, recent research presents inconsistent results in this regard (Hodge et al., 2018; Kannangara et al., 2018; Schmidt et al., 2018; Frontini et al., 2021; Lam and Zhou, 2022). This study confirmed that grit is associated with higher OP based on data analysis. We also found how two potential moderators (i.e., SC and TL) can strengthen the relationship between grit and OP; however, there has been limited research on the role of grit in performance (Credé et al., 2017; Khan et al., 2020). To be specific, when the organization consists of gritty employees, it is likely to show a higher performance level. Additionally, when the organization has a supportive organizational climate and is led by transformational leaders, the organization is expected to show a strong relationship between grit and performance.

### Theoretical Contributions

This study makes a theoretical contribution by broadening the understanding of the benefits of grit. Research so far has focused on the micro-level impact of grit on things like career success, academic performance, and athletic performance (Credé et al., 2017; Arco-Tirado et al., 2019; Arifin et al., 2019; Lam and Zhou, 2019). This study, however, examined macro-level performance, results on which are limited in the management discipline. When an organization is made up of hardworking individuals, it can expect higher levels of firm-level performance. Thus, this research adds to management studies. Second, grit is an important trait, especially when an organization is facing a crisis (Kannangara et al., 2018; Rego et al., 2021). This paper suggests how an organization can survive a crisis by demonstrating the relationship between grit and performance when the focal company faces a major crisis such as the COVID-19 pandemic (Budhwar and Cumming, 2020; Nguyen et al., 2022). This study contributes to the literature by suggesting the requisite personal traits for tough times (Rego et al., 2021). Third, while the importance and effectiveness of grit have been studied, little attention has been paid to contingency and the expression of grit (Credé et al., 2017; Jordan et al., 2019; Choi et al., 2020; Khan et al., 2020). Therefore, this study adds to the literature by looking at how grit can be expressed based on organizational climate and leadership.

### Practical Implications

The results of this study have several implications for organizational psychological practice in terms of personnel selection and development. First, the human resource department needs to be aware of employees' potential (Kannangara et al., 2018) and reflect on the types of human capital to be recruited in the organization as well as the traits that must be developed in them. Thus, grit can be an important factor for organizations for personnel selection and development (Jordan et al., 2019; Luthans et al., 2019). Second, as gritty employees contribute to the organization, the HR department can create a supportive climate through policies and practices to increase the employees' effectiveness (Collins and Smith, 2006; Kim et al., 2021). Furthermore, TL characteristics, such as idealized influence, intellectual stimulation, inspirational motivation, and individualized consideration (Bass and Avolio, 2000), should be demonstrated by leaders to enhance organizational performance (Bakker et al., 2022). HR managers must select and promote transformational leaders and develop them through training, coaching, and feedback programs (Bruch and Walter, 2007).

### Limitations and Future Research

Despite the aforementioned contributions, this study has several theoretical, technical, and analytical limitations. First, we used TAT as an overarching theory to explain trait-performance relationships. A recent review study examined situational features such as functions, organizational culture, leadership, and personal motivation (Tett et al., 2021). Because this study looked at the moderating effects of organizational climate and leadership, future research could look into the impact of personal characteristics and functions on organizational performance. Second, because we gathered the data from a single source, the common method variance may have caused some bias when we calculated the associations between variables (Gardner III and Schermerhorn, 1992). A single-factor test may indicate whether a single factor explains all the covariances among the items, but it does not statistically adjust for method effects (Podsakoff et al., 2003). The normative effect of a single organizational culture could have reduced grit variance (Podsakoff et al., 2003). To generalize our findings to a larger population, future researchers should use multiple sources to demonstrate the given correlations after accounting for both random and fixed variables. Third, because all participants are nested within the same group (i.e., company A), they may exhibit similar attitudes and behaviors (Sacco et al., 2003). Given the data's hierarchically nested structure, future research should focus on multilevel analysis (Hofmann and Gavin, 1998) techniques such as hierarchical linear modeling (Kassinis and Vafeas, 2006) and multilevel structural equation modeling (Ryu and West, 2009). Finally, because the response rate appears to be low in the study, increasing the response rate is recommended for future studies to enhance the value of research findings (Baruch and Holtom, 2008).

## Conclusion

This study emphasizes the importance of developing and managing hardworking employees, creating a supportive climate, and fostering transformational leadership—all of which can lead to higher organizational performance. Organizations should be able to create a supportive climate, select and develop transformational leaders based on individual grit, and thus improve their performance. This study adds to the literature on sustainable business in crisis by demonstrating the interaction effects of individual traits, organizational climate, and leadership.

## Data Availability Statement

The raw data supporting the conclusions of this article will be made available by the authors, without undue reservation.

## Ethics Statement

The studies involving human participants were reviewed and approved by Gachon University Institutional Review Board. The patients/participants provided their written informed consent to participate in this study.

## Author Contributions

The author confirms being the sole contributor of this work and has approved it for publication.

## Conflict of Interest

The author declares that the research was conducted in the absence of any commercial or financial relationships that could be construed as a potential conflict of interest.

## Publisher's Note

All claims expressed in this article are solely those of the authors and do not necessarily represent those of their affiliated organizations, or those of the publisher, the editors and the reviewers. Any product that may be evaluated in this article, or claim that may be made by its manufacturer, is not guaranteed or endorsed by the publisher.
